# Integrated Transcriptomic and Metabolomic Analysis Reveals the Meat Production Features in Hybrid Sheep

**DOI:** 10.3390/ani16010137

**Published:** 2026-01-03

**Authors:** Zhenghan Chen, Shuwei Dong, Liwa Zhang, Xuejiao An, Qiao Li, Zhenfei Xu, Zhiguang Geng, Haina Shi, Chune Niu, Rui Zhang, Yaojing Yue

**Affiliations:** 1 Key Laboratory of Animal Genetics and Breeding on the Tibetan Plateau, Ministry of Agriculture and Rural Affairs, Lanzhou Institute of Husbandry and Pharmaceutical Sciences, Chinese Academy of Agricultural Sciences, Lanzhou 730050, China; 19139107648@163.com (Z.C.); 17899319024@163.com (L.Z.); anxuejiao@caas.cn (X.A.); liqiao@caas.cn (Q.L.); chuneniu@163.com (C.N.); 2 Sheep Breeding Engineering Technology Research Center of Chinese Academy of Agricultural Sciences, Lanzhou 730050, China; 3 Shaanxi Provincial Engineering and Technology Research Center of Cashmere Goats, Life Science Research Center, Yulin University, Yulin 719000, China; dongshuwei2005@126.com; 4 Institute of Agricultural Sciences of Qingyang, Qingyang 745000, China; 236xuzhenfei@163.com (Z.X.); gengzhiguang2@163.com (Z.G.); shihaina0716@163.com (H.S.)

**Keywords:** sheep, Suffolk, Hu sheep, meat production, heterosis, multi-omics

## Abstract

This study was conducted to investigate potential regulatory mechanisms of hybridization increased the meat production performance in sheep. Thirty-six 3-month-old male lambs of Suffolk sheep (SFK, *n* = 12), Hu sheep (HH, *n* = 12), and their F_1_ hybrids (SH, *n* = 12) were selected and raised in individual pens under identical nutritional supply and husbandry management regimes over a 95-day (including a 15-day pre-trial period) experimental period. At the end of the feeding trial, six sheep closest to the average body weight were selected from each group for the subsequent trial, involving the collection of Longissimus dorsi samples and the determination of production performance, muscle fiber characteristic and transcriptomic and metabolomic analysis. The results showed that the SH sheep had significantly higher pre-slaughter live weight and carcass weight than the HH sheep, while lower than those of the SFK sheep (*p* < 0.05). The muscle fiber density of the SH group was significantly higher than that of the parental groups, while the muscle fiber diameter and cross-sectional area were significantly smaller (*p* < 0.05). The collagen fiber content of the SH group was intermediate between the two parental groups and significantly higher than that of the SFK group (*p* < 0.05). Transcriptomic analysis identified 2920 differentially expressed genes (DEGs), which were mainly enriched in the AMPK, PI3K-Akt, and PPAR signaling pathways. Metabolomic analysis detected 1617 differential metabolites (DMs), which were enriched in the fatty acid degradation and steroid hormone biosynthesis pathways. Integrated analysis revealed that core genes *SESN3* and metabolites (malate, testosterone) enhance energy supply capacity through AMPK pathway, thereby promoting muscle fiber proliferation and increasing meat yield in the hybrid sheep. In conclusion, the heterosis of the SH group originates from the remodeling of muscle fiber structure and the synergistic regulation of related pathways, which provides a theoretical basis for sheep crossbreeding.

## Simple Summary

Mutton is prized by consumers for its high protein content; however, domestic production remains insufficient to meet market demand. Crossbreeding is an effective approach to enhance production performance and meat quality in hybrid offspring. To elucidate the regulatory mechanisms underlying this heterosis, this study compared the meat yield and muscle fiber characteristics of Hu sheep, Suffolk sheep, and their F_1_ hybrids. Through transcriptomic and metabolomic analyses of the M. Longissimus dorsi thoracis et lumborum (hereafter referred to as Longissimus dorsi) muscle, we identified key differentially expressed genes (DEGs) and differential metabolites (DMs). This aims to construct a molecular regulatory network for heterosis in meat production, thereby providing novel targets for sheep breeding.

## 1. Introduction

The global demand for animal protein continues to increase annually, it is estimated to rise by 38% over the period from 2020 to 2050 [1]. Sheep is prized by consumers for its high protein content. China is the foremost sheep-raising nation globally, possessing the largest herds and highest annual slaughter figures. This capacity solidifies its position as the world leader in both mutton output and consumption. However, despite this scale, domestic production remains insufficient to satisfy the total demand. Improving mutton yield remains a top priority for animal husbandry science and technology workers. Crossbreeding can integrate the excellent genes of different parent breeds, enabling the offspring to exhibit more superior traits than the parents [2]. Crossbreeding remains a core technology in modern agricultural production and plays an irreplaceable role in improving the flesh quality and overall productivity of livestock products. Hu sheep is a multi-fetal sheep breed in China, characterized by high fecundity, good parenting traits, and strong adaptability. However, it also has shortcomings such as slow growth rate, low meat production performance, and unsatisfactory meat-type body conformation. Suffolk is a prominent meat sheep breed originating in England. Suffolk sheep have been introduced to China for over 30 years, and targeted acclimatization measures—such as crossbreeding with local breeds and adjustments in feeding strategies—have been widely implemented. Research confirms that both purebred Suffolk sheep and their crossbred offspring exhibit strong adaptability to the temperate continental climate of northern China, where the study was conducted, showing no significant differences in growth performance, survival rates, or disease resistance compared to local breeds [3]. It is known for its rapid lamb growth rates, substantial musculature, superior dressing percentage, and high lean meat yield. In recent years, it has been widely employed as a terminal sire in crossbreeding programs across China. Numerous studies have reported that crossbreeding can improve the production performance and meat quality of hybrid offspring in animals. The average daily gain of F_1_ hybrids from Texel and Segureña sheep increased by 12% compared to the female parent and by 18% compared to the male parent, while the muscle protein content increased by 2.1% [4]. The F_1_ hybrids produced by crossing Mongolian dam with Small-tailed Han ewes are superior to the female parent in growth performance [5]. Compared to their purebred parents, the crossbred offspring of Aardi and Damascus goats demonstrate enhanced heat tolerance coupled with superior production performance [6]. In general, crossbreeding can improve the growth rate and meat production performance in sheep, while the regulatory mechanism underlying the resulting heterosis remains unclear.

Recent advances in omics technologies have provided novel insights for illuminating the regulatory mechanisms underlying heterosis. Specifically, transcriptome sequencing (RNA-seq) permits the high-throughput exploration of key pathways and candidate genes affecting muscle quality, thereby facilitating the analysis of gene regulatory networks [7]. And it has been widely used to study the molecular mechanisms underlying important economic traits in animals [8,9]. As the end products of gene expression and intermediary metabolism, metabolites provide a direct readout of the biochemical phenotype of muscle. The application of metabolomics in meat science is therefore particularly powerful, owing to its inherent advantages of high sensitivity, ability to capture dynamic processes, and strong correlation with observable meat quality traits. The integrated analysis of transcriptomics and metabolomics can capture the dynamic changes in gene expression and metabolites to reveal the multi-level regulatory network of muscle quality formation, overcoming the limitations of single omics. It is thus an effective approach for investigating the genetic mechanism of heterosis. Studies have shown that differentially expressed genes (DEGs) between hybrid individuals and purebred parents may drive phenotypic advantages by regulating pathways related to metabolism, immunity, and cell proliferation [10,11]. In the hybrid offspring of Dorper × Mongolian sheep, the *MYF5* and *IGF1* genes in muscle tissue are significantly upregulated, which are closely associated with the growth and development of muscle fibers [12]. In immune-related tissues, the activation of the TLR4/MyD88 pathway may enhance the anti-infection ability of hybrid sheep [13]. Metabolomics, on the other hand, can accurately capture the dynamic changes in metabolites in key pathways such as lipid metabolism (e.g., glycerophospholipid metabolism, fatty acid biosynthesis), amino acid synthesis, and flavor substance formation [14]. Extensive research indicates that omics-based integrated analysis combines the differences and complementarities between the two omics studies to clarify gene expression patterns and regulatory processes. It has been widely applied to decipher the molecular mechanisms underlying the differences in production performance between hybrid offspring and their parents [15,16].

This study selected Hu sheep as the female parent, Suffolk as the male parent, and their F_1_ hybrid offspring as research objects, starting with the comparative analysis of meat yield and muscle fiber characteristics among the three groups. Through transcriptomic and metabolomic sequencing combined with integrated analysis, this study screened for differentially expressed genes (DEGs) and differential metabolites (DMs) in the longissimus dorsi muscle between the F_1_ hybrid sheep and their parents, aiming to construct a molecular regulatory network for the heterosis of meat production performance, and provide novel targets for the breeding of new sheep breeds.

## 2. Materials and Methods

### 2.1. Animals, Diets, and Experimental Design

This study was conducted at the Gansu Qinghuan meat sheep seed production co., LTD located in Huanxian County, Gansu, China. The male parents of Suffolk sheep (SFK) used in this study were descendants of breeding rams imported from Australia in 2018. This strain is renowned for its large body size, rapid growth rate, and outstanding meat production performance. In contrast, the female parents of Hu sheep (HH) were selected from the core conservation population in Jiangsu region, which is characterized by excellent reproductive performance (year-round estrus and high fecundity) and good environmental adaptability. Clarifying this parental combination with a relatively distant genetic distance not only helps to explain the heterosis exhibited by the hybrid offspring but also provides a clear genetic background for subsequent pedigree-based molecular marker-assisted breeding. The experimental design, procedures, and methodologies employed in this study have been previously published [17] and was approved by the Lanzhou Institute of Husbandry and Pharmaceutical Sciences, Chinese Academy of Agricultural Sciences Institutional Animal Care and Use Committee under the permit NO.2024-13 following the Chinese Standards for the use and care of research animals.

The feeding trial in this study employed a one-factor completely randomized experimental design, with crossbreeding type as the core fixed-effect factor, and three experimental groups were established. Thirty-six 3-month-old male lambs of Suffolk (SFK, *n* = 12), Hu sheep (HH, *n* = 12) and their hybrid F_1_ generation (SH, *n* = 12) with the birth date were selected. They were individually housed under identical nutritional supply and husbandry management regimes over a 95-day (including a 15-day pre-trial period) experimental period, feeding a complete mixed pellet diet and ad libitum feeding and watering throughout the experiment. The diet was formulated according to the feeding objectives, and the nutrient levels on a dry matter basis were calculated based on the China Feed Composition and Nutrient Value Table [18]. The feed formula is presented in Table 1.

Six lambs close to the average body weight per group at the end of the experiment were selected and humanely sacrificed. All animals were fasted for 12 h with free access to water. Then based on halal procedure, sheep were individually restrained, exsanguinated, peeled and eviscerated. Approximately 1 cm × 2 cm × 0.5 cm longissimus dorsi from the carcass left side between 12 and 13th rib was collected by a scalpel within 30 min post-slaughter and fixation in 4% paraformaldehyde for muscle staining, another two portions 2 g longissimus dorsi samples were collected and transferred into a sterile tube, immediately frozen using liquid nitrogen, then stored at −80 °C for DNA and metabolite extraction.

### 2.2. Muscle Tissue Staining

After fixation, the samples were dehydrated in a graded anhydrous ethanol solution (Sigma-Aldrich, St. Louis, MO, USA), embedded in paraffin (Leica Biosystems, Buffalo Grove, IL, USA, HistoGel™), and then sectioned. After dewaxing, the sections were processed using H&E staining (hematoxylin–eosin staining). The stained sections were observed under an upright microscope (Nikon, ci-s, Tokyo, Japan). Slide Viewer 2.6 software (3DHISTECH, Budapest, Hungary) was used to scan the section images at 20× magnification to observe their morphological characteristics, and Image J software (v1.8.0) was employed to measure the diameter, area, and density of muscle fibers [17].

For masson staining, paraffin sections were first dewaxed and hydrated, then stained with Weigert’s iron hematoxylin (Electron Microscopy Sciences, Hatfield, PA, USA) solution for 5 min, rinsed with running water, and subjected to bluing treatment, subsequently, stained with ponceau-acid fuchsin (Electron Microscopy Sciences, Hatfield, PA, USA) solution for 5 min and rinsed with running water, treated with phosphomolybdic acid solution (Sigma-Aldrich, USA) for approximately 5 min. After removing excess liquid, the sections were counterstained with aniline blue (Leica Biosystems, Tokyo, Japan, Masson) for 3 min, followed by rinsing with 1% acetic acid aqueous (Sigma-Aldrich, USA) solution until no blue color was released from the sections, rinsed with 95% alcohol (Sigma-Aldrich, USA), dehydrated with anhydrous ethanol and graded xylene, and finally mounted with neutral resin. The mounted sections were observed under an upright microscope (Nikon, ci-s, Japan). Slide Viewer 2.6 software (3DHISTECH, Hungary) was used to scan the section images at 20× magnification to observe morphological characteristics, and Image J software (v1.8.0) was used to determine the relative content of collagen fibers [17].

### 2.3. Transcriptome Sequencing and Bioinformatics Analysis

Polyadenylated mRNA was isolated from total RNA using mRNA Capture Beads. The purified mRNA was subsequently fragmented by exposure to high temperature. Using this fragmented mRNA as a template, first-strand cDNA was synthesized in a reverse transcription reaction. Subsequently, during the second-strand synthesis step, the resulting double-stranded cDNA underwent end repair and received a 3′ dA-overhang. Adaptors were then ligated to these A-tailed fragments, and the final library was purified and size-selected using Hieff NGS^®^ DNA Selection Beads. PCR library amplification is then performed, and finally, detection is carried out using the Illumina Novaseq X Plus (Illumina, San Diego, CA, USA). To obtain high-quality clean reads, the reads were further filtered using fastp (version 0.18.0). The short-read alignment tool Bowtie2 (version 2.2.8) was used to map reads to the ribosomal RNA (rRNA) database. The rRNA-mapped reads were then removed. The remaining clean reads were further used for assembly and gene abundance calculation. Then the genomic DNA sequence file (in FASTA format) can be obtained from the link: https://ftp.ensembl.org/pub/release-115/fasta/ovis_aries_gca022416695v1/dna/ accessed on 8 December 2024, using HISAT2 2.1.0 [19]. The mapped reads of each sample were assembled using StringTie v1.3.1. For each transcription region, an FPKM (fragment per kilobase of transcript per million mapped reads) value was calculated to quantify its expression abundance and variations using RSEM software 1.3.3 [20]. RNA differential expression analysis was performed using DESeq2 between two different groups [21]. FDR < 0.05 and |log2FC| ≥ 2 were considered differential expressed genes. Gene Ontology (GO) and Kyoto Encyclopedia of Genes and Genomes (KEGG) pathway enrichment analyses of the DEGs were performed using R based on hypergeometric distribution. GO terms and KEGG pathways with *p* < 0.05 were considered significantly enriched.

### 2.4. Metabolomic Sequencing and Bioinformatics Analysis

A 100 mg sample was taken and 1 mL of cold 90% methanol was added, homogenized, centrifuged and re-dissolved in 100 μL of acetonitrile/water (1:1, *v*/*v*) solvent for LC-MS analysis.

Chromatographic separation was performed on an ACQUIY UPLC BEH Amide column (2.1 mm × 100 mm, 1.7 µm; Waters, Ireland) using a UHPLC system (1290 Infinity LC, Agilent Technologies Santa Clara, CA, USA) coupled to a quadrupole time-of-flight mass spectrometer (AB Sciex TripleTOF 6600, AB Sciex, Framingham, MA, USA). The mobile phase for both ESI positive and negative modes consisted of solvent A (25 mM ammonium acetate and 25 mM ammonium hydroxide in water) and solvent B (acetonitrile). The gradient elution program was set as follows: 85% B was maintained for 1 min, linearly decreased to 65% B over 11 min, rapidly reduced to 40% B in 0.1 min and held for 4 min, then returned to 85% B in 0.1 min, followed by a 5 min re-equilibration period. The ESI source conditions were configured with Ion Source Gas 1 and 2 at 60, curtain gas at 30, a source temperature of 600 °C, and an IonSpray Voltage Floating of ±5500 V. Data were acquired in the range of 25–1000 Da using an information-dependent acquisition (IDA) method, with a product ion scan accumulation time of 0.05 s/spectra. Following acquisition, raw data were normalized for metabolite annotation and quantification after quality control assessment, which included monitoring the clustering of QC samples to evaluate data stability [22].

Orthogonal partial least squares discriminant analysis (OPLS-DA) was used to distinguish the differences in metabolic profiles between the three experimental groups. Differential metabolites (DMs) were screened by setting a double threshold: variable importance projection (VIP) score ≥ 1 and statistical significance level *p* < 0.05. Subsequently, metabolic pathway enrichment analysis was performed based on the annotation information of the KEGG database.

### 2.5. Quantitative Real-Time PCR (qRT-PCR) Analysis

A subset of differentially expressed genes (DEGs) was selected at random for validation via quantitative real-time polymerase chain reaction (qRT-PCR). Primer sequences for the target genes are provided in Table S1, with the GAPDH gene serving as the internal reference for normalization. Total RNA was isolated from samples using an RNA extraction kit purchased from Xavier Biotechnology Co., Ltd. (Wuhan, China), while first-strand cDNA synthesis was implemented with a reverse transcriptase kit from the same manufacturer. All qRT-PCR assays were conducted on a Bio-Rad fluorescent quantitative PCR system. The thermal cycling protocol included an initial pre-denaturation step at 95 °C for 30 s, followed by 40 amplification cycles; each cycle comprised 15 s of denaturation at 95 °C, 30 s of annealing at 60 °C, and a 10-s extension phase at 72 °C. To confirm amplification specificity, a melting curve analysis was subsequently performed over a temperature range of 65 °C to 95 °C, with fluorescence signal acquisition occurring at 0.5 °C increments. Relative transcriptional levels of each gene were ultimately determined using the 2^−∆∆CT^ calculation method.

### 2.6. Statistical Analysis

Prior to statistical analyses, data were screened for normality and outliers using SPSS 27.0 (IBM Corp. International Business Machines Corporation, Armonk, NY, USA). One-way ANOVA, supplemented with Tukey’s post hoc test for significant results, was applied to analyze slaughter performance, carcass traits, muscle fiber characteristics, and gene expression. Significant correlations between differentially expressed genes (DEGs) and differential metabolites (DMs) were defined as |r| > 0.6 with *p* < 0.05 (Pearson). GraphPad Prism 8.0 was utilized for graph generation, and all analyses were based on six biological replicates.

## 3. Results

### 3.1. Slaughter Performance and Carcass Traits

The slaughter performance and carcass traits of the SFK, SH, and HH groups were conducted (Table 2). The pre-slaughter live weight, carcass weight, loin eye area, bone weight, and meat weight of the SH group were significantly lower than those of the SFK group and significantly higher than those of the HH group (*p* < 0.05). The dressing percentage and GR value in the SH and SFK groups were significantly higher than the HH group (*p* < 0.05). The backfat thickness of the SH and HH groups was significantly lower than the SFK sheep (*p* < 0.05). In terms of organ weight indices, the head, skin and liver weight of the SH group were significantly lower than these of the SFK group and higher than these of the HH group (*p* < 0.05). The spleen, lung and kidney weight of the SH group were significantly higher than these of the HH group (*p* < 0.05), and there was no significant difference between the SH and SFK groups. The hoof weight of the SH group was significantly lower than that of the SFK groups, while the heart weight was significantly lower than that of the HH and SFK groups (*p* < 0.05).

### 3.2. Muscle Fiber Characteristics Analysis

**Figure 1:**
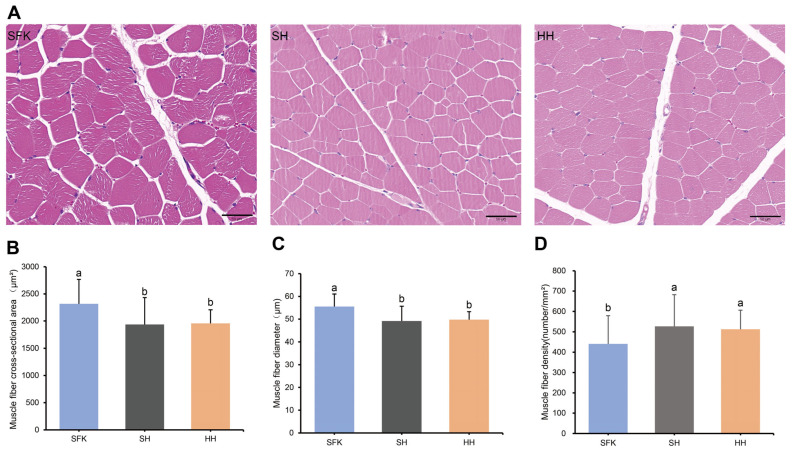
Morphological characteristics of muscle fibers in SFK, SH, and HH groups. (**A**) HE-stained sections of longissimus dorsi muscle tissue. (**B**–**D**) Comparison of muscle fiber cross-sectional area, muscle fiber diameter, and muscle fiber density among the three groups. All data are presented as mean ± standard deviation (*n* = 6). Different letters indicate significant differences (*p* < 0.05).

The HE staining showed that the cell nuclei were stained blue, and the muscle fibers were stained red (Figure 1A). A significant reduction in muscle fiber cross-sectional area and diameter was observed in the SH and HH groups compared to the SFK group. In contrast, the muscle fiber density index showed an opposite trend, being significantly higher in the SH and HH groups (Figure 1B–D, *p* < 0.05).

**Figure 2:**
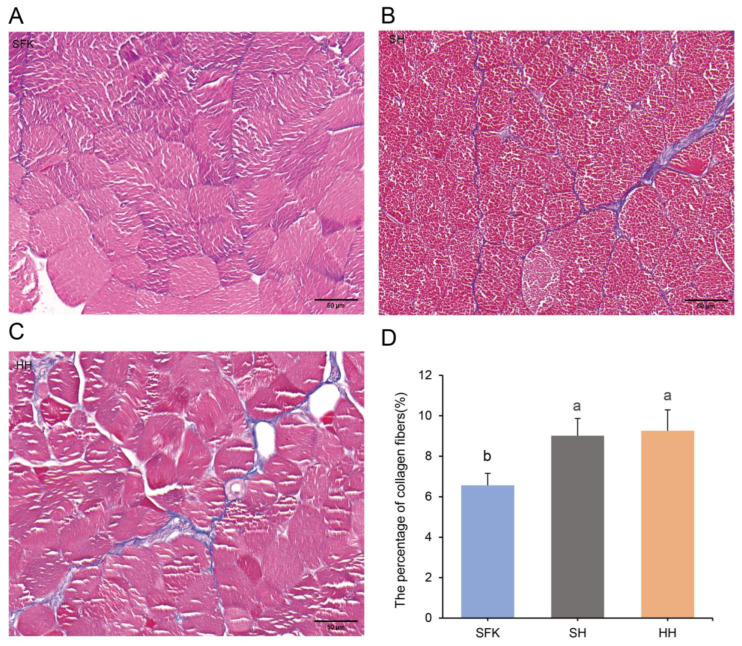
Detection of collagen fiber content in the longissimus dorsi muscles of SFK, SH, and HH groups using Masson staining. (**A**–**C**) Masson-stained sections of longissimus dorsi muscle tissue. (**D**) Comparison of collagen fiber content among the three groups. All data are presented as mean ± standard deviation (*n* = 6). Different letters indicate significant differences (*p* < 0.05).

The Masson staining was used to determine the content of collagen fibers in the three groups. Collagen fibers, mucus, and cartilage were stained blue, muscle fibers, cellulosic substances, and red blood cells appeared red, and cell nuclei were stained bluish-black (Figure 2A–C). Significant differences in muscle collagen fiber content were revealed by quantitative analysis (Figure 2D). The SFK sheep exhibited the lowest proportion, while the HH sheep showed the highest; the SH sheep occupied an intermediate position. The collagen content of the SH group was significantly higher than that of the SFK group (*p* < 0.05).

### 3.3. Transcriptomic Analysis

**Figure 3:**
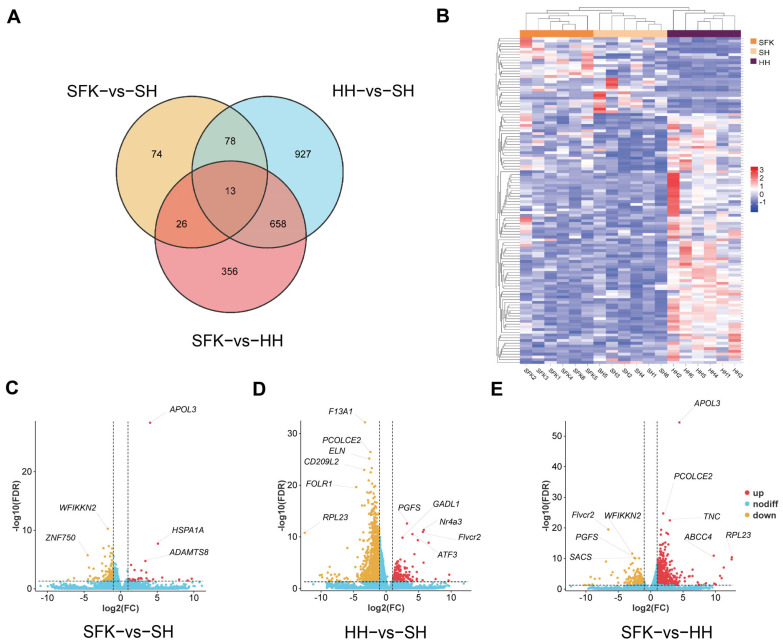
Transcriptome comparison of longissimus dorsi muscles between SFK and SH group, HH and SH group, SFK and HH group. (**A**) Venn diagram of DEGs. (**B**) Clustering heatmap of DEGs. (**C**–**E**) Volcano plot of differentially expressed genes (DEGs).

After filtering and quality control of the raw data, the three groups obtained an average of 290,421,517 high-quality clean reads. The overall high quality of the sequencing data, suitable for subsequent in-depth analyses, was evidenced by several key metrics: Q30 values between 91.11% and 95.38%, a mean GC content of 52.95%, a low sequencing error rate of 0.01%, and a mapping rate exceeding 83.33% for all samples (Table S2). A total of 2920 DEGs were identified among the three groups, according to the Venn diagram of DEGs (Figure 3A), there are 13 common DEGs in all animals, including *C4BPA*, *FOLR2*, *CSF1R*, *MYH13*, *ATP6V0A4*, *Nr4a3*, *TMEM255A*, *SACS*, *MSTN*, *FLVCR2*, *ACBD3*. And the heatmap of DEGs demonstrated clear segregation among the groups (Figure 3B). Differentially expressed genes (DEGs) were screened using |log2Fold Change| ≥ 1 and FDR < 0.05 as criteria (Table S3). The volcano plot indicated that 160 annotated DEGs (25 upregulated and 135 downregulated genes) were obtained by comparing the SFK with SH groups, among which the significantly upregulated differential genes included *APOL3*, *HSPA1A*, and *ADAMTS8*, while *WFIIKKN2* and *ZNF750* were the top five among the significantly downregulated ones (Figure 3C). A total of 1559 annotated DEGs (163 upregulated and 1396 downregulated genes) were identified when we compared the HH and SH groups, with the significantly upregulated differential genes being *PGFS*, *GADL1*, *Nr4a3*, *ATF3*, and *Fivcr2* and the significantly downregulated differential genes including *F13A1*, *PCOLCE2*, *ELN*, *CD209L2*, *FOLR1*, and *RPL23* (Figure 3D). Additionally, 936 annotated DEGs (729 upregulated and 207 downregulated genes) were obtained by comparing the SFK with HH groups, where the significantly upregulated differential genes were *APOL3*, *PCOLCE2*, *TNC*, *ABCC4*, and *RPL23*, and the significantly downregulated differential genes included Fivcr2, *WFIKKN2*, *PGFS*, and *SACS* (Figure 3E). Furthermore, we found that *MSTN* gene, associated with muscle growth, presented a higher trend in the SFK group than HH and SH groups. *HK2* gene, which regulates glucose phosphorylation, was significantly increased in the SFK group than the SH group.

**Figure 4:**
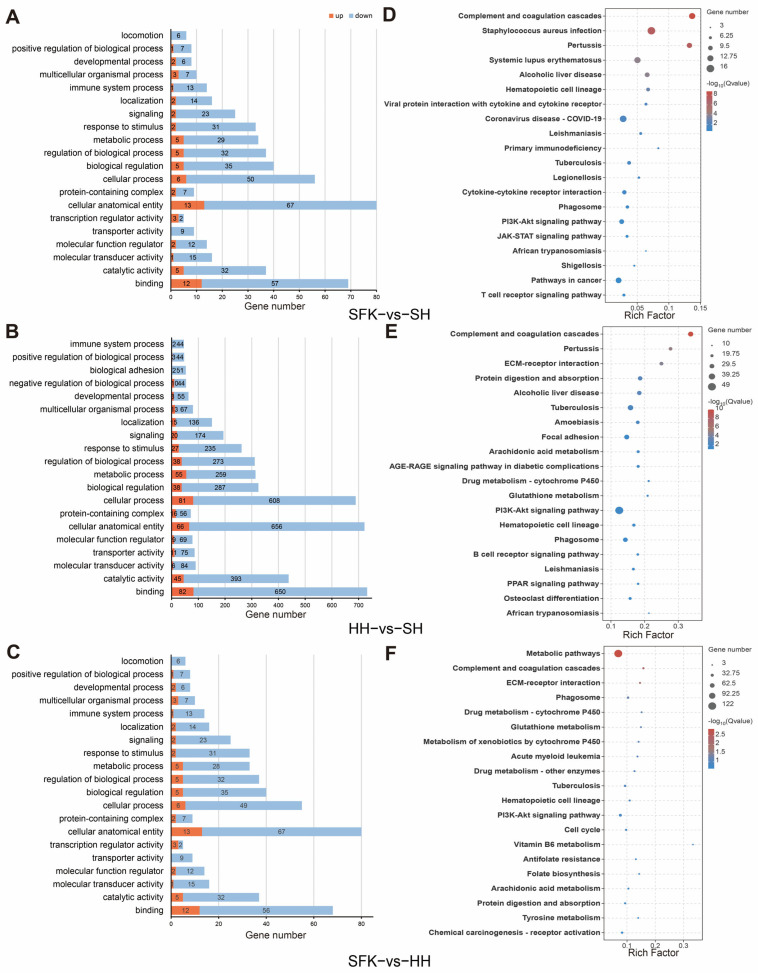
Functional enrichment analysis of longissimus dorsi samples. (**A**) GO enrichment analysis of SFK vs. SH groups. (**B**) GO enrichment analysis of HH vs. SH groups. (**C**) GO enrichment analysis of SFK vs. SH groups. (**D**) KEGG enrichment analysis of SFK vs. SH groups. (**E**) KEGG enrichment analysis of SFK vs. SH groups. (**F**) KEGG enrichment analysis of SFK vs. SH groups.

To gain a more comprehensive understanding of the functional characteristics of the selected differentially expressed genes (DEGs), we performed GO and KEGG functional enrichment analyses. GO analysis showed that the DEGs between the SFK and SH groups were significantly enriched in 33 GO terms (*p* < 0.05, Figure 4A, Table S4), among which 299 genes were related to biological processes (BP), 89 to cellular components (CC), and 161 to molecular functions (MF). At level 2, they were mainly enriched in cellular anatomical entity, binding and cellular process. The DEGs between the HH and SH groups were significantly enriched in 42 GO terms, including 2667 genes related to BP, 111 to CC, and 1565 to MF (Figure 4B, Table S4). And they were mainly concentrated in “collular procass”, “catalytic activity”, “binding” and “cellular anatomical entity”. When comparing the SFK and HH groups, there were 33 GO terms significantly enriched, with 297 genes related to BP, 89 to CC, and 249 to MF (Figure 4C, Table S4). Among these, the DEGs were mainly enriched in pathways regulating energy supply such as response to stimulus and regulation of biological process, metabolic process, biological regulation, cellular process, and cellular anatomical entity.

KEGG enrichment analysis showed that 30 signaling pathways were significantly enriched when comparing the SFK with the SH group (Figure 4D, Table S5), among which DEGs were mainly enriched in the complement and coagulation cascades, staphylococcus aureus infection and pertussis. Moreover, AMPK and PI3K-Akt signaling pathway were related to energy metabolism and muscle growth and development also enriched in the SFK group. In the comparison between the HH and the SH group, 52 pathways were significantly enriched (Figure 4E, Table S5), with DEGs mainly enriched in the complement and coagulation cascades, pertussis and ECM–receptor interaction. For the SFK vs. HH group, analysis showed that 40 pathways exhibited significant enrichment (Figure 4F, Table S5), among which DEGs were mainly enriched in the metabolism pathways.

### 3.4. Metabolomic Analysis

**Figure 5:**
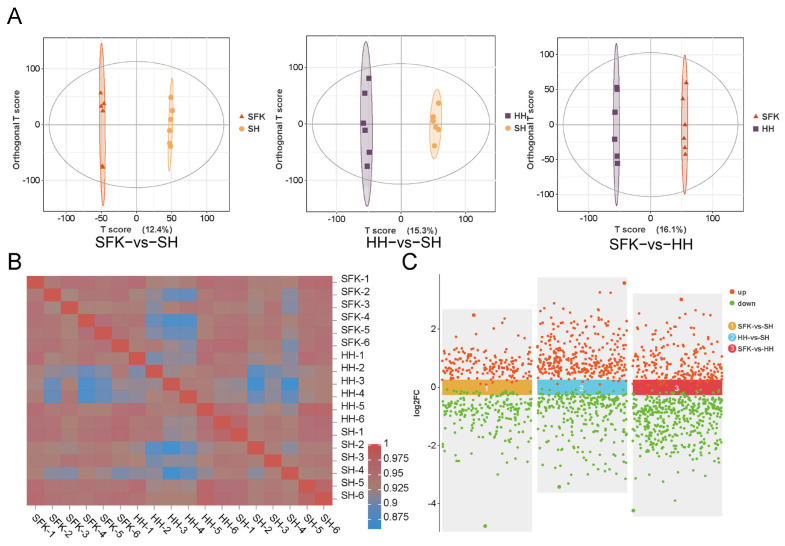
(**A**) OPLS-DA analysis of SFK vs. SH groups, HH vs. SH groups, SFK vs. HH groups. (**B**) The inter-sample correlation heatmap. (**C**) Scatter plot for SFK vs. SH groups, HH vs. SH groups, SFK vs. HH groups.

To investigate the metabolic differences in the longissimus dorsi muscle, we conducted an LC-MS/MS analysis, detecting a total of 1114 metabolites (Table S6). Orthogonal partial least squares-discriminant analysis (OPLS-DA) unveiled significant differences in the metabolic profiles among the three groups (Figure 5A). The inter-sample correlation heatmap also confirmed this (Figure 5B). Differential metabolites (DMs) were selected using a threshold of variable importance in projection (VIP) ≥ 1 and a *p*-value < 0.05. A total of 373 DMs were identified between the SFK and SH groups, among which 170 DMs were upregulated and 203 DMs were downregulated. The comparative analysis of the HH and SH groups revealed 591 DMs comprising 328 upregulated and 263 downregulated. A total of 653 DMs were detected between the SFK and HH groups, with 219 being upregulated and 434 being downregulated. Then, the selection of the top 15 differential metabolites was performed using the VIP value as the primary criterion. Rimsulfuron, Pyrifenox, 2-Naphthalenesulfonic acid, Thiacloprid, Cefazolin, Naloxone hydrochloride, 3α,7α-Dihydroxy-12-oxocholanoic acid, Mevalonic acid, 17β-Nandrolone decanoate, 2′Hydroxy4′methoxyacetophenone, 3-Hydroxy-4-methoxycinnamic acid, Diethyl phthalate, Fenthion, D-Galacturonic acid, L-2-Hydroxyglutaric acid.

**Figure 6:**
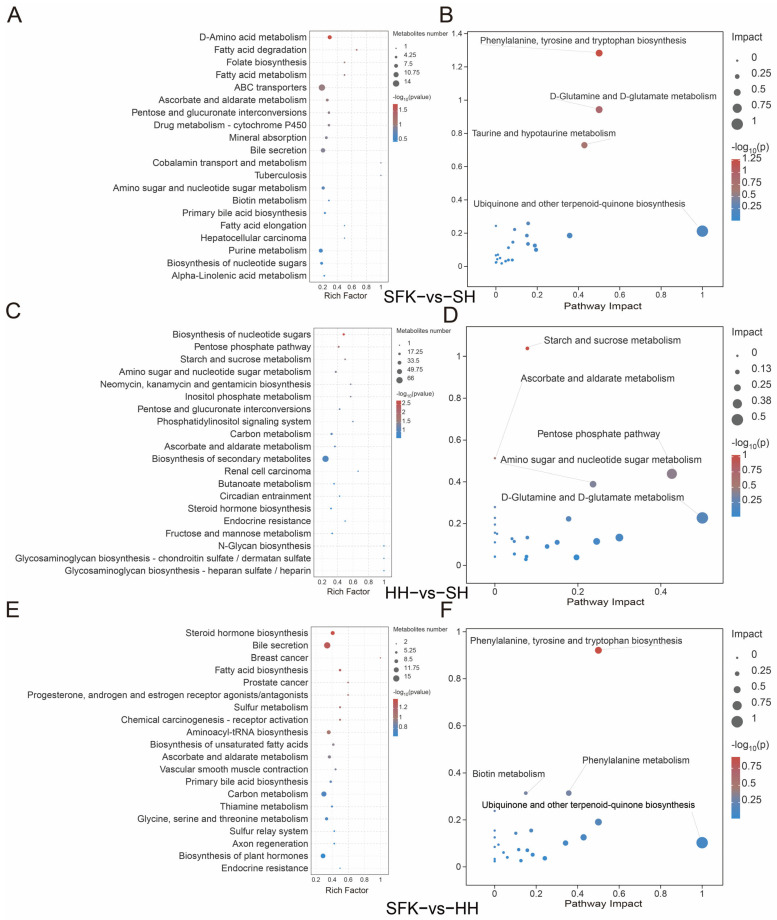
(**A**) KEGG enrichment bubble plot between the SFK and SH groups. (**B**) MetPA enrichment plot between the SFK and SH groups. (**C**) KEGG enrichment bubble plot between the HH and SH groups. (**D**) MetPA enrichment plot between the HH and SH groups. (**E**) KEGG enrichment bubble plot between the SFK and HH groups. (**F**) MetPA enrichment plot between the SFK and HH groups.

According to the KEGG pathway enrichment analysis, 98 DMs were enriched in 115 pathways between the SFK and SH groups, among which most DMs were enriched in fatty acid degradation and D-amino acid metabolism pathways (Figure 6A, Table S7). MetPA analysis was performed, demonstrating that the phenylalanine, tyrosine, and tryptophan biosynthesis pathway and the D-glutamine and D-glutamate metabolism pathway were crucial within the regulatory network (Figure 6B, Table S8). When comparing the HH and SH groups, 163 DMs were enriched in 170 pathways. Most of these DMs were enriched in the nucleotide sugar biosynthesis, pentose phosphate pathway, starch and sucrose metabolism, amino sugar and nucleotide sugar metabolism, biosynthesis of neomycin, and inositol phosphate metabolism (Figure 6C, Table S7). The MetPA analysis revealed that the starch and sucrose metabolism pathway and the pentose phosphate pathway played a pivotal role in the regulatory network (Figure 6D, Table S8). In the SFK and HH groups, a total of 168 DMs were enriched in 169 pathways, among which most differential metabolites were enriched in steroid hormone biosynthesis and bile secretion pathways (Figure 6E, Table S7). Integrated with the MetPA results, it was through our pathway enrichment analysis that the biosynthesis of aromatic amino acids (phenylalanine, tyrosine, and tryptophan) and the catabolism of phenylalanine were delineated as being significantly enriched, as essential components of the regulatory network (Figure 6F, Table S8).

### 3.5. Integrated Analysis of Transcriptome and Metabolome

**Figure 7:**
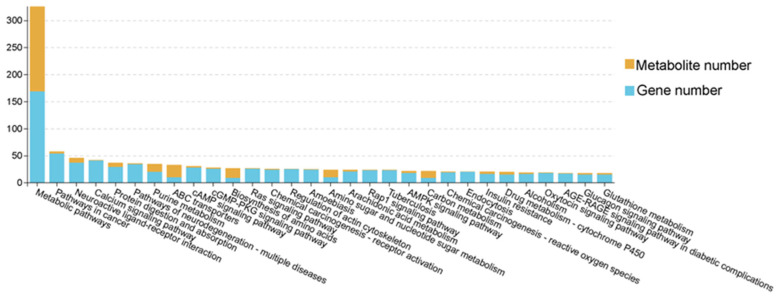
Column charts of pathway association analysis between SFK group and SH group, HH group and SH group, and SFK group and HH group.

**Figure 8:**
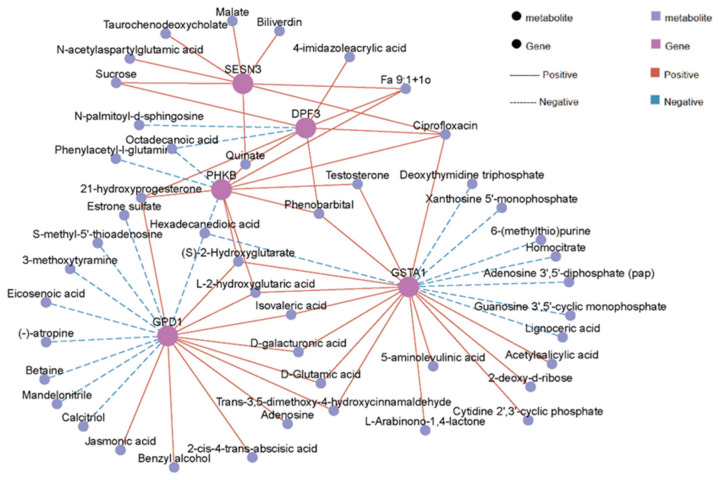
Correlation network diagram of differentially expressed genes (DEGs)–differential metabolites (DMs).

Our integrated analysis of transcriptomic and metabolomic data has revealed 161 significantly enriched KEGG pathways, mainly including metabolic pathways, purine metabolism, protein digestion and absorption, biosynthesis of amino acids and AMPK signaling pathway (Figure 7, Table S9). Furthermore, Pearson correlation analysis identified significantly correlated DEG–DM pairs (|r| > 0.6, *p* < 0.05, Figure 8, Table S10). These associations were mainly enriched in biological pathways such as energy metabolism (glycolysis, tricarboxylic acid cycle), signal transduction (AMPK, PI3K-Akt) and lipid metabolism (PPAR signaling pathway). Among them, energy metabolism and lipid metabolism pathways showed the highest enrichment. Specifically, *SESN3* gene, an energy stress-sensing protein, is positively related to malate, which is an intermediate metabolite produced in the tricarboxylic acid cycle. D-galacturgnic acid and D-Glutamic acid were positively associated with *GSTA1* and *GPD1* genes.

### 3.6. qRT-PCR Validation of Functional Gene Expression

**Figure 9:**
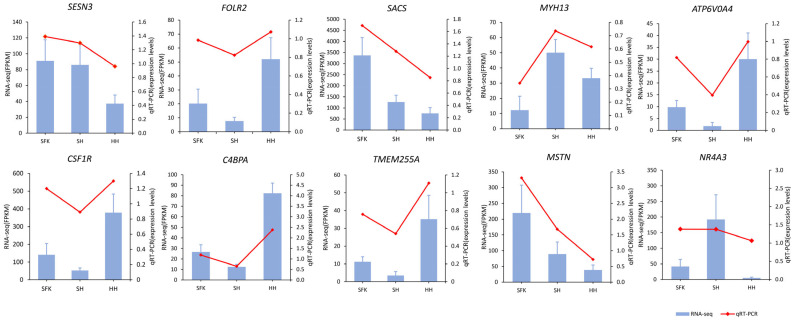
Validation of differentially expressed genes by RT-qPCR.

To validate the transcriptomic data, 10 differentially expressed genes (DEGs) were randomly selected, and their mRNA levels were assessed using quantitative real-time PCR (qRT-PCR). The expression patterns of these genes aligned with the RNA-seq data (Figure 9), thereby validating the reliability of our transcriptomic findings.

## 4. Discussion

Slaughter performance and carcass traits are key indicators for evaluating the muscle growth and development of animals. In this study, the SH group showed lower values than the SFK group in indices directly related to muscle growth and development, including pre-slaughter live weight, dressing percentage, carcass weight, loin eye area, bone weight, meat weight, backfat thickness, and GR value, while presented a higher value than the purebred Hu sheep, which indicting that the SH exhibit heterosis in muscle growth and development compared with their female parents. This result is also consistent with our previous study; the Polled Dordet × Hu hybrid F_1_ generation exhibited significantly superior meat production performance compared to the purebred Hu sheep [23]. In the present study, the meat–bone ratio of the SH group was significantly lower than that of the SFK and HH groups. Crossbreeding may have affected the growth and development dynamics of muscle and bone tissues, resulting in a relative delay in the peak of muscle tissue deposition compared with bone tissue at the end of the feeding trial. The development and function of visceral organs directly determine metabolic capacity of animals, and the weight and size of visceral organs serve as a direct reflection, thereby influencing meat production performance [24]. In this study, the weight of most visceral organs was significantly higher in the SH group than the Hu group, implying that the SH group possesses a more robust digestive and metabolic capacity, which in turn promotes animal growth and development. This further confirms that the SH group has heterosis in muscle growth and development compared with the HH group, but it does not exhibit such heterosis in this aspect when compared with the SFK group.

This study compared the muscle fiber characteristics and collagen fiber content between hybrid offspring and their parents. The number and size of muscle fibers determine the outcome of muscle growth and the characteristics of muscle fibers, including their density, diameter, cross-sectional area, as well as collagen fiber content, are direct determinants of meat quality [25,26]. In this study, the cross-sectional area and diameter of fibers in SH group was significantly lower than that of the SFK group and showing no significant difference from the HH group, conversely, the SH group had a higher density than both the SFK and HH groups. This may be attributed that the muscle fibers of the hybrid SH group undergo structural remodeling during growth and development, in other word, the cross-sectional area and diameter of muscle fibers decrease simultaneously, the density of muscle fibers increases significantly, and the phenotype of muscle fibers deviates from the mean value of the two parents. Following the completion of normal growth and development of muscle tissue, the quantity of skeletal muscle fibers essentially undergoes no further alterations, and the late stage of muscle growth and development is mainly characterized by muscle fiber hypertrophy and type transformation. This “high density-small diameter” phenotype suggested that crossbreeding may promote muscle growth and development by activating the muscle fiber hyperplasia pathway rather than the traditional hypertrophy pathway [27,28]. Fibrous collagen is dominant in the subendothelial matrix, serving as a key component actively involved in the component makeup of this layer, at the same time, it serves as the primary connective tissue protein within animal organisms [29]. Studies have shown that collagen content and collagen cross-linking play key roles in meat tenderness. The content of collagen fibers in muscle is negatively correlated with tenderness, whereas the content of heat-soluble collagen fibers in muscle is positively correlated with meat tenderness [30,31]. This study, a markedly greater total collagen fiber content was observed in the HH and SH groups compared to the SFK group, suggesting this disparity as a potential contributor to the variations in meat quality.

Analysis of differentially expressed genes (DEGs) among the three groups showed the high expression of the *HK2* gene in the SH group may accelerate the conversion of glucose to glucose-6-phosphate, providing more substrates for glycolysis, thereby increasing ATP production and providing energy support for muscle fiber proliferation (such as the increased muscle fiber density in the SH group). Myostatin (*MSTN)*, also known as growth differentiation factor 8 (GDF-8), is a member of the transforming growth factor-β (TGF-β) superfamily and a glycoprotein widely expressed in the body [32]. Studies have shown that the deletion of the *MSTN* gene affects intestinal barrier function through the MLCK/p-MLC signaling pathway, affecting the composition of the intestinal microbiota, and further promotes the growth of fast-twitch glycolytic muscles by increasing valeric acid production, and valeric acid activates the Akt/mTOR pathway through the SCFAs receptor GPR43 [33]. In this study, the expression level of the *MSTN* gene in the SH group was significantly lower than that in both the SFK group and the HH group (*p* < 0.05), which is consistent with the phenotypic data of carcass weight among the three groups of experimental sheep. The DEGs between the SFK and the SH groups are mainly enriched in responses to organic substances and external stimuli, forming a stress defense network, cell periphery and extracellular space, multicellular organism development, system development, and regulation of immune system processes. The co-enrichment of multicellular development genes and immune regulatory genes reflects a resource trade-off strategy—under stress, muscle fiber proliferation is inhibited, and immunity is prioritized for activation [34]. Studies have found that extracellular receptors sense stress signals and activate the AMPK pathway, which coordinates “defense-first” metabolic reprogramming by inhibiting mTORC1 [35]. The coordinated activity of the AMPK and PI3K/AKT signaling pathways orchestrates key physiological processes in skeletal muscle, including myogenesis and the regulation of fatty acid metabolism. Studies have shown that activation of the PI3K/AKT signaling pathway promotes myocyte survival and maintains muscle fiber homeostasis, which is consistent with the function of the ECM–receptor interaction pathway in porcine skeletal muscle development [36,37]. When muscle cells are damaged, extracellular receptors sense and activate AMPK and PI3K/Akt, which inhibit muscle fiber differentiation and upregulate immune effects, leading to adaptive muscle remodeling [38]. For the DEGs between the HH group and the SH group, the KEGG enrichment analysis identified several significantly enriched pathways. These included metabolic pathways such as arginine and proline metabolism and inositol phosphate metabolism, alongside signaling and energy pathways like the AMPK signaling pathway and oxidative phosphorylation. The PPAR pathway and regulation of lipolysis in adipocytes synergistically enhance fatty acid β-oxidation through the PPARγ-PGC1α axis, providing energy for muscle fiber hypertrophy. When muscles are subjected to mechanical stimulation, plasma membrane receptors are activated to trigger Ca^2+^ release, leading to ATP-dependent kinase phosphorylation. This causes the PPAR pathway to regulate the expression of genes involved in lipid metabolism in the liver and skeletal muscle, thereby promoting muscle fiber hypertrophy [39,40]. Transcriptomic analysis in this study revealed that when the Suffolk crossed with Hu sheep, although genes related to meat production were downregulated in the SH group, genes related to meat quality were upregulated.

Our study utilized a metabolomics approach to identify and quantify the differential metabolites across the various groups. Significant enrichment of specific KEGG pathways was observed based on the differential metabolites, highlighting key metabolic distinctions between the SFK and SH treatments, the differential metabolic pathways involved D-amino acid metabolism and fatty acid degradation. Studies have shown that the synergistic effect of such pathways can reshape the material and energy metabolism patterns of muscle cells, affecting energy supply and structural stability of muscle fibers [41,42]. Between the HH and SH groups, differences pathways were enriched in the nucleotide sugar biosynthesis and pentose phosphate pathway, which were associated with carbohydrate metabolism. By regulating glycogen metabolism and redox balance, these pathways indirectly affect physiological states of muscles [43,44]. Between the SFK and HH groups, differences in pathways such as steroid hormone biosynthesis and fatty acid biosynthesis synergize with muscle growth and differentiation signaling pathways, interfering with muscle fiber type transformation and developmental processes, thereby being associated with muscle growth and development [45].

Based on the integrated analysis of transcriptome and metabolome, this study conducted an in-depth analysis of the molecular synergistic patterns regulating muscle physiological functions, thereby providing crucial insights into the mechanisms underlying muscle energy metabolic homeostasis and quality formation. In the tricarboxylic acid (TCA) cycle, a core pathway of energy metabolism, significant inter-group differences were observed in the key intermediate metabolite malate, which exhibited a significant positive correlation with the expression of the regulatory gene *SESN3*. As an energy stress-sensing protein, high expression of *SESN3* can accelerate the metabolic flux involving malate: on one hand, it promotes the production of NADH and FADH_2_, providing substrates for mitochondrial ATP synthesis and ensuring energy supply to muscle cells [46,47]; on the other hand, this process synergizes with the enrichment of the AMPK signaling pathway. As an energy-sensing molecule, AMPK coordinates the balance between energy metabolism and muscle cell functions by regulating the transcription of TCA cycle-related genes, precisely matching energy supply with consumption in response to changes in muscle cell energy demand [48]. The AMPK signaling pathway was significantly enriched among groups, and the core gene *SESN3* was deeply associated with various energy metabolism-related metabolites such as malate and succinate derivatives, enabling muscle energy sensing and metabolic adaptation through the AMPK/mTOR pathway [49]. *SESN3* can sense changes in the intracellular ATP/AMP ratio, activate AMPK, and inhibit mTOR signaling to reduce unnecessary energy consumption. Associated metabolites act as “energy signal carriers,” and their content changes synergize with SESN3 expression to precisely regulate metabolic pathway flux, maintaining muscle cell energy homeostasis and physiological functions [47,48,49,50].

Expression of *PHKB* and *DPF3* showed a positive correlation with testosterone levels. This correlation was found to be under the transcriptional regulation of the PI3K-Akt signaling pathway [51]. Enrichment of the PI3K-Akt pathway can activate steroid synthesis genes and promote testosterone production. Testosterone promotes satellite cell proliferation and muscle fiber hypertrophy through the androgen receptor (AR), and can also enhance protein synthesis via the mTOR pathway, maintaining muscle strength and function, thus providing a complete “hormone-signal-function” axis for muscle growth and quality maintenance [52].

The differential genes, metabolites, and pathways between the SH sheep and its parents contributed to the phenotypic differences in muscle growth and development, and this represented the main molecular mechanisms underlying the heterosis of the SH sheep.

## 5. Conclusions

In this study, we systematically compared muscle fiber density, diameter, cross-sectional area, and collagen content between hybrid offspring and their parents. Integrated transcriptomic and metabolomic analysis revealed that the *SESN3* gene activates the AMPK signaling pathway and modulates *HK2* expression, thereby enhancing glycolytic metabolism. This increase promotes the generation of malate and other tricarboxylic acid cycle intermediates for energy supply and further facilitate muscle fiber hypertrophy, and ultimately lead to superior meat yield of hybrid sheep. Taken together, The hybrid offspring have achieved a synergistic balance among growth performance, environmental adaptability, and breeding benefits, making them an excellent and well-suited choice for local sheep producers. Meanwhile, these findings provide new insights into elucidating the molecular mechanisms underlying sheep muscle growth and development as well as the formation of heterosis in hybrid offspring.

## Figures and Tables

**Table 1:** Composition of Total Mixed Ration and Its Nutritional Levels on a Dry Matter Basis.

Ingredients	Content	Nutrient Level	Content
Corn	40.00	Dry Matter (DM)	88.68
Soybean meal	6.00	Crude Protein (CP)	15.88
Wheat bran	4.50	Ether Extract (EE)	3.08
Cottonseed meal	4.60	Crude Ash (Ash)	7.24
Corn germ meal	5.30	Metabolic Energy (MJ/kg)	11.65
Malt root	5.00	Acid Detergent Fiber (ADF)	16.99
DDGS	6.00	Neutral Detergent Fiber (NDF)	31.50
Molasses	3.50	Calcium (Ca)	0.97
Straw meal pellet	20.00	Phosphorus (P)	0.49
Sodium bicarbonate	1.00		
Calcium carbonate	1.20		
200 slow-release urea	0.50		
Sodium chloride	0.60		
Calcium hydrogen phosphate	0.50		
Magnesium oxide	0.10		
Yeast culture	0.50		
1% premix	0.70		
Total	100.00		

Content of 1% Premix: Cu: 1300 mg/kg, Fe: 6000 mg/kg, Mn: 4500 mg/kg, Zn: 8500 mg/kg, I: 170 mg/kg, Se: 45 mg/kg, Co: 65 mg/kg, Vit A 800 KIU/kg, Vit D3 350 KIU/kg, Vit E 7000 IU/kg.

**Table 2:** Slaughter performance and carcass traits of SFK, HH and SH groups.

Items	SFK	SH	HH
Slaughter performance			
Pre-slaughter live weight/kg	71.23 ± 3.99 ^a^	59.00 ± 4.68 ^b^	52.63 ± 3.48 ^c^
Dressing percentage/%	55.58 ± 0.78 ^a^	54.82 ± 2.67 ^a^	49.61 ± 1.42 ^b^
Carcass traits			
Carcass weight/kg	40.83 ± 2.93 ^a^	34.47 ± 2.76 ^b^	25.67 ± 0.82 ^c^
Loin eye area/cm^2^	33.45 ± 3.57 ^a^	26.25 ± 2.16 ^b^	18.43 ± 0.91 ^c^
Bone weight/kg	9.53 ± 1.23 ^a^	8.9 ± 0.07 ^b^	6.03 ± 0.62 ^c^
Meat weight/kg	31.04 ± 2.72 ^a^	25.7 ± 2.30 ^b^	19.53 ± 1.18 ^c^
Meat-to-bone ratio	3.26 ± 0.57 ^a^	2.9 ± 0.24 ^b^	3.3 ± 0.52 ^a^
Backfat thickness/mm	5.9 ± 1.10 ^a^	4.2 ± 1.54 ^b^	4.2 ± 0.42 ^b^
GR value/mm	12.1 ± 1.14 ^a^	13.0 ± 2.30 ^a^	11.4 ± 0.26 ^b^
Organs weight			
Head weight/kg	3.13 ± 0.15 ^a^	2.76 ± 0.12 ^b^	2.29 ± 0.17 ^c^
Hoof weight/kg	1.76 ± 0.21 ^a^	1.28 ± 0.05 ^b^	1.16 ± 0.14 ^b^
Skin weight/kg	5.96 ± 0.62 ^a^	5.23 ± 0.23 ^b^	4.67 ± 0.27 ^c^
Heart weight/kg	0.28 ± 0.06 ^a^	0.22 ± 0.03 ^b^	0.30 ± 0.06 ^a^
Liver weight/kg	1.26 ± 0.09 ^a^	1.08 ± 0.15 ^b^	0.91 ± 0.09 ^c^
Spleen weight/kg	0.12 ± 0.03 ^a^	0.10 ± 0.01 ^a^	0.07 ± 0.01 ^b^
Lung weight/kg	0.91 ± 0.12 ^a^	0.89 ± 0.12 ^a^	0.58 ± 0.10 ^b^
Kidney weight/kg	0.18 ± 0.03 ^a^	0.19 ± 0.02 ^a^	0.13 ± 0.01 ^b^

In the same row of data, different letters indicate significant differences. (*p* < 0.05). Data are presented as mean ± standard deviation (*n* = 6).

## Data Availability

The original data presented in the study are openly available in the NCBI Sequence Read Archive (SRA) database (PRJNA1337443).

## Supplementary Materials

The following supporting information can be downloaded at: https://www.mdpi.com/article/10.3390/ani16010137/s1. Table S1: Details of primer sequences of miRNAs used for qRT-PCR; Table S2: Statistics of RNA-Seq data quality; Table S3: Differentially expressed genes in longissimus dorsi; Table S4: GO Enrichment Analysis of Differentially Expressed Genes Among SFK, SH, and HH Groups; Table S5: KEGG enrichment analysis of Differentially Expressed Genes Among SFK, SH, and HH Groups; Table S6: Differential metabolites of longissimus dorsi among SFK, SH, and HH Groups; Table S7: KEGG analysis of differential metabolites among SFK, SH, and HH Groups; Table S8: MetPA analysis of differential metabolites Among SFK, SH, and HH Groups; Table S9: Pathway Association Analysis; Table S10: Pairs of Differentially Expressed Genes and Differential Metabolites.
