# Supplementary material for: Integrated Transcriptomic and Metabolomic Analysis Reveals the Meat Production Features in Hybrid Sheep
Source: Animals (Basel). 2026 Jan 3;16(1):137. doi: 10.3390/ani16010137 (PMC12784912; doi:10.3390/ani16010137)
Supplement: Supplementary file 1 [file animals-16-00137-s001.zip › Table S2.pdf]

Supplementary Table S2. Statistics of RNA-Seq data quality

Sample	Raw Data(bp)	Clean Data(bp)	Q20(%)	Q30(%)	N(%)	GC(%)
SFK1	7707293700	7593544578	7389702772 (97.32%)	7058447577 (92.95%)	58331 (0.00%)	3985389131 (52.48%)
SFK2	5968602600	5783266573	5639447414 (97.51%)	5376645598 (92.97%)	977481 (0.02%)	2958614951 (51.16%)
SFK3	7838899500	7562963431	7322724642 (96.82%)	6890818562 (91.11%)	526394 (0.01%)	4087892340 (54.05%)
SFK4	6692838000	6476160075	6287003564 (97.08%)	5967450079 (92.14%)	188514 (0.00%)	3419228872 (52.80%)
SFK5	7554346200	7295559985	7097494086 (97.29%)	6740902596 (92.40%)	171247 (0.00%)	3870461896 (53.05%)
SFK6	7302159300	7047414974	6857153415 (97.30%)	6512046792 (92.40%)	168526 (0.00%)	3748998086 (53.20%)
SH1	7360842600	7084223898	6909153247 (97.53%)	6576178908 (92.83%)	158380 (0.00%)	3756513309 (53.03%)
SH2	5679672900	5497376855	5355912907 (97.43%)	5100930430 (92.79%)	953998 (0.02%)	2947973155 (53.63%)
SH3	6369963600	6120427792	5916782738 (96.67%)	5591255979 (91.35%)	2160552 (0.04%)	3246312527 (53.04%)
SH4	5567820000	5372782543	5213506741 (97.04%)	4938031210 (91.91%)	164651 (0.00%)	2815088857 (52.40%)
SH5	5744461500	5519146948	5351404137 (96.96%)	5073757223 (91.93%)	1923603 (0.03%)	2925086121 (53.00%)
SH6	6560036700	6395602632	6231793007 (97.44%)	5927427858 (92.68%)	152467 (0.00%)	3415566881 (53.40%)
HH1	7291806000	7070518645	6887140084 (97.41%)	6548965346 (92.62%)	169476 (0.00%)	3786663767 (53.56%)
HH2	7441499100	7148661745	6943254148 (97.13%)	6575324980 (91.98%)	172265 (0.00%)	3710969427 (51.91%)
HH3	5769377100	5576289956	5432278651 (97.42%)	5176583495 (92.83%)	398946 (0.01%)	2939078551 (52.71%)
HH4	8284001700	8103970863	7976099897 (98.42%)	7729623611 (95.38%)	499993 (0.01%)	4314414589 (53.24%)
HH5	12245644500	11927375397	11660145794 (97.76%)	11155639102 (93.53%)	469129 (0.00%)	6343675657 (53.19%)
HH6	9310417800	9079398748	8833349354 (97.29%)	8415864255 (92.69%)	1891443 (0.02%)	4836515364 (53.27%)

Raw Data(bp): Total base pairs of raw sequencing data (unit: bp) directly output by the sequencer.

Clean Data(bp): Total base pairs of high-quality filtered data (unit: bp) after processing raw data.

Q20 (%): Count and percentage of bases with sequencing quality score ≥Q20 relative to Clean Data.

Q30 (%): Count and percentage of bases with sequencing quality score ≥Q30 relative to Clean Data.

N (%): Count and percentage of single-end reads containing N bases (undetermined base) relative to Clean Data.

GC (%): Proportion of guanine (G) and cytosine (C) in filtered sequences (Clean Data).
